# Tumors Sharply Increased after Ceasing Pazopanib Therapy for a Patient with Advanced Uterine Leiomyosarcoma: Experience of Tumor Flare

**DOI:** 10.1155/2017/4801650

**Published:** 2017-04-06

**Authors:** Terumi Tanigawa, Shintaro Morisaki, Hisanobu Fukuda, Shuichiro Yoshimura, Hisayoshi Nakajima, Kohei Kotera

**Affiliations:** Department of Obstetrics and Gynecology, Nagasaki Harbor Medical Center City Hospital, 6-39 Shinchimachi, Nagasaki-shi, Nagasaki 850-8555, Japan

## Abstract

Pazopanib has activity in patients with soft-tissue sarcoma. We report an advanced uterine leiomyosarcoma case that suddenly worsened after cessation of pazopanib therapy. A 47-year-old woman had a primary uterine leiomyosarcoma tumor and multiple lung metastases, which progressed during her initial treatment. In subsequent treatment with pazopanib for 3 months, the sum of her tumor diameters after cessation sharply increased for two weeks. Symptoms such as dyspnea suddenly worsened also. She died of the disease one month after cessation of pazopanib therapy. Given the poor prognosis of recurrent uterine leiomyosarcoma and the rapid tumor enlargement after ending pazopanib therapy, control of this disease is especially important. Therefore, the decision to discontinue pazopanib therapy requires careful consideration.

## 1. Introduction

Uterine leiomyosarcomas (LMS) are associated with poor prognosis, with an average five-year survival rate of around 40% [1, 2]. Moreover, median overall survival after recurrence is under 12 months [3, 4].

The treatment for unresectable advanced or recurrent uterine LMS is chemotherapy. Standard first-line chemotherapy has been doxorubicin with or without ifosfamide [5]. The response rate of this combination chemotherapy was reported as complete response in 3% to 16% of patients and partial response in 27% to 32% of patients [6, 7].

If the disease does not respond to standard chemotherapy, one agent of interest is pazopanib because of its activity in patients with soft-tissue sarcoma [8]. Pazopanib has been comprehensively defined as a synthetic indazolpyrimidine with activity as a small-molecule vascular endothelial growth factor (VEGF) inhibitor (specifically, as a multitargeted tyrosine kinase inhibitor) against VEGFs 1, 2, and 3 and platelet-derived growth factors. Pazopanib was approved as a second-line treatment after a phase III trial of this drug reported a statistically significant increase in progression-free survival [9, 10]. Pazopanib has been available in Japan for treating soft-tissue sarcoma since 2012.

Many tyrosine kinase inhibitors (TKIs) are also used in the treatment of other cancers such as non-small cell lung cancer, chronic myeloid leukemia, renal cell carcinoma, hepatocellular carcinoma, and thyroid cancer. Recently, several reports have demonstrated cases of rapid tumor size increase related to cessation of TKI treatment [11–15].

In this report, we present an advanced LMS case that suddenly worsened after cessation of pazopanib therapy.

## 2. Case Presentation

A 47-year-old woman (nullipara) with no past history was diagnosed with uterine LMS of FIGO stage IVB, with multiple lung, liver, and bone metastases. We retrospectively reviewed the medical records of the patient so as to assess the outcomes and adverse events of therapy. We used the Response Evaluation Criteria in Solid Tumors (ver 1.1) to assess tumor responses and the Common Terminology Criteria for Adverse Events (ver 4.0) to assess adverse events. The growth modulation index (GMI), the ratio of time to progression (TTP) with present therapy and TTP with previous therapy, is calculated as follows: GMI = TTP_present therapy_/TTP_previous therapy_ [16]. We calculated the GMI, ratio of TTP after discontinuing pazopanib to TTP while receiving chemotherapy (docetaxel with gemcitabine, adriamycin, and pazopanib).

The patient had symptoms of genital bleeding, and a uterine tumor was identified. Findings from a tumor biopsy were suggestive of uterine LMS. The patient was treated with neoadjuvant chemotherapy (docetaxel with gemcitabine) for two courses. However, the primary tumor, peritoneal dissemination, and the lung metastases progressed. The attending physician indicated surgery in order to reduce tumor volume and to confirm diagnosis by pathology. Subsequently, she was treated with surgical resection (total abdominal hysterectomy with bilateral salpingoophorectomy and partial omentectomy). Pathological examination confirmed the uterine LMS diagnosis and the presence of metastatic tumors in both ovaries and the omentum. After resection, the patient underwent chemotherapy (adriamycin) for three courses; although lung metastases became stable, peritoneal dissemination progressed. As we judged the patient's disease to have progressed as a whole, we decided to cease the adriamycin treatment. Subsequently, we treated the patient with pazopanib therapy (800 mg) orally once daily for three months. Prior to commencing pazopanib therapy, her echocardiography findings were normal. The patient did not experience pazopanib therapy-related adverse events at severe grades but did develop grade II hypertension that responded to antihypertensive medication. A CT scan taken three months after starting pazopanib treatment showed that the tumor sizes of the liver and lung metastases and of the peritoneal dissemination were stable (Figure 2). However, pazopanib therapy was discontinued due to the new occurrence of a metastasis to the skin. At this time, we began considering a different type of chemotherapy.

**Figure 1:**
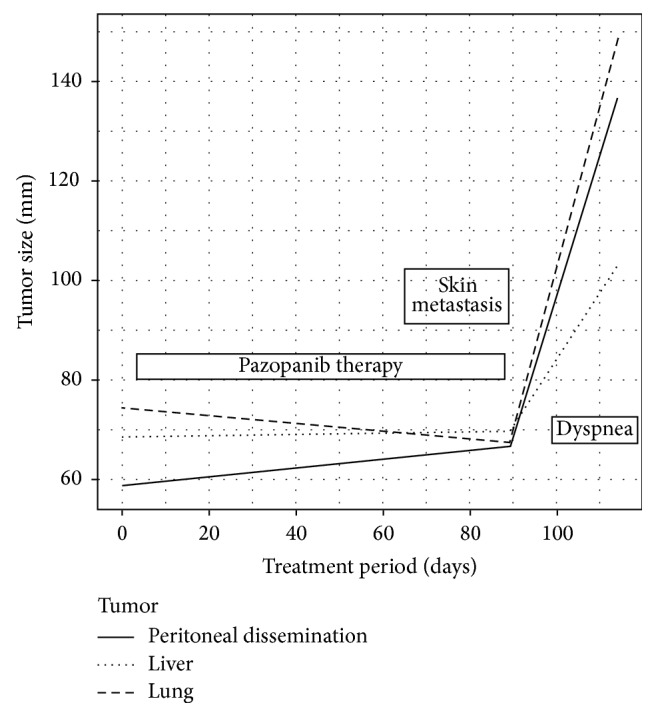
The graph shows the change of tumor size with course of treatment. The sum of the patient's tumor diameters increased after cessation of pazopanib therapy.

**Figure 2:**
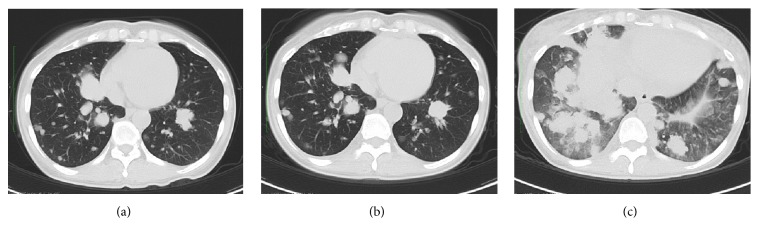
Transverse section of lung in computed tomography. (a) Start of pazopanib therapy. (b) End of pazopanib therapy. (c) After two weeks' cessation of pazopanib therapy.

After ceasing pazopanib therapy, cough symptoms sharply increased for two weeks. Her echocardiography findings were again normal. Additionally, the sum of the patient's tumor diameters increased as follows: for lung metastases, by −10% (during the 3-month-pazopanib treatment) versus by 55% (during the two weeks after ceasing treatment); for liver metastases, by 0% versus by 33%; and for peritoneal dissemination, by 12% versus by 51% (Figure 1). Particularly, the lung metastases sharply increased (Figure 2). The GMI was 0.18 (ratio of TTP after discontinuing pazopanib to TTP while receiving chemotherapy).

The patient also experienced sudden worsening of symptoms, such as severe dyspnea which was difficult to control. She had an emergency hospitalization for the severe dyspnea. Large enlargement of the right ventricle and dysfunction of the left ventricle were confirmed by echocardiography. A CT scan did not show any evidence of pulmonary infarction or pulmonary bleeding. Therefore, the patient was diagnosed with acute pulmonary heart. The dyspnea was treated with morphine and oxygen administration; however, the patient's symptoms did not improve. The patient died after four days under sedation. She had died one month after cessation of the pazopanib therapy.

## 3. Discussion

A global, double-blind, randomized, phase III trial of pazopanib for metastatic soft-tissue sarcoma compared pazopanib once daily versus placebo as second-line or later treatment for patients with advanced soft-tissue sarcoma [10]. In this trial, progression-free survival was significantly improved in the pazopanib arm (median, 4.6 versus 1.6 months; hazard ratio, 0.31; *P* < .001). On the basis of the results of the trial, pazopanib is currently recommended as one of the treatments for patients with advanced soft-tissue sarcoma after failure of standard chemotherapy.

We experienced pazopanib therapy for an advanced or recurrent uterine LMS patient whose condition suddenly worsened after cessation of pazopanib therapy. The sum of the tumor diameters after cessation sharply increased for two weeks. Furthermore, this patient's sudden tumor size increase after ceasing pazopanib therapy appeared related to uncontrolled severe dyspnea.

Some reports have claimed that the cessation of TKI therapy has been followed by the rapid progression of what is termed “flare-up” or “tumor flare” [11–15]. The mechanism for this phenomenon has not been clarified. One hypothesis is the rapid growth of TKI-sensitive clones following the discontinuation of the drug [12, 13]. Another possible explanation is that the residual inhibitory effects of the antiangiogenics disappear after cessation of therapy [14]. One such study found that some (23%) patients with EGFR-mutant lung cancer and acquired resistance to TKIs experienced disease flare after discontinuation of TKI and that the median time to flare was 8 days (range 3–21 days) [12]. Another study of tumor flare occurrence and its prognostic role after discontinuing anti-VEGF receptor TKIs investigated patients with metastatic renal cell carcinoma, similarly concluding that TKI discontinuation accelerates tumor growth rate and negatively affects prognosis [15]. Chaft et al. have reported that shorter time to progression on initial TKI treatment and the presence of pleural or central nervous system metastases are associated with tumor flares in patients with lung cancer [12].

The growth modulation index (GMI) is the ratio of time to progression (TTP) with present therapy and TTP with previous therapy. It has been suggested that GMI > 1.33 indicates a drug or drug combination is active [16]. In our patient, the GMI was 0.18 (ratio of TTP after discontinuing pazopanib to TTP while receiving pazopanib). This GMI suggests that her disease progressed suddenly after discontinuing pazopanib therapy.

A phase II study has shown that trabectedin is a useful therapeutic agent [17] and this new agent was recently approved for treatment of advanced soft-tissue sarcoma; however, we could not use it to treat our patient because it had not yet been approved.

Some studies have suggested that continuing some therapies beyond identification of progressive disease can be clinically beneficial; this is termed “beyond PD” [18, 19]. The concept of beyond PD may be relevant to avoidance of “flare-up” or “tumor flare” associated with TKI therapy.

If the patient above had continued pazopanib therapy, such symptoms might have instead been mild. Therefore, when considering this case for sudden tumor growth after ending pazopanib treatment and for absence of adverse events of severe grade, it appears that treatment could have been further continued in light of comparative risks of disease progression. The prognosis of recurrent uterine leiomyosarcomas is poor; thus, disease control is important.

## 4. Conclusion

In conclusion, on the basis of our experience conducting pazopanib therapy for patients with advanced LMS, we conclude that the decision to discontinue pazopanib therapy requires careful consideration.
